# Detection and characterization of *Wolbachia* infections in laboratory and natural populations of different species of tsetse flies (genus *Glossina*)

**DOI:** 10.1186/1471-2180-12-S1-S3

**Published:** 2012-01-18

**Authors:** Vangelis Doudoumis, George Tsiamis, Florence Wamwiri, Corey Brelsfoard, Uzma Alam, Emre Aksoy, Stelios Dalaperas, Adly Abd-Alla, Johnson Ouma, Peter Takac, Serap Aksoy, Kostas Bourtzis

**Affiliations:** 1 Department of Environmental and Natural Resources Management, University of Ioannina, 2 Seferi St, 30100 Agrinio, Greece; 2 Yale University School of Public Health, 60 College St., 811 LEPH, New Haven, CT 06520, USA; 3 Current address: Department of Entomology, University of Kentucky, S-225 Ag. Science Center North, Lexington, KY 40546, USA; 4 Insect Pest Control Laboratory, Joint FAO/IAEA Division of Nuclear Techniques in Food and Agriculture, Vienna, Austria; 5 Trypanosomiasis Research Centre, Kenya Agricultural Research Institute, P.O. Box 362, Kikuyu 00902, Kenya; 6 Institute of Zoology, Section of Molecular and Applied Zoology, Slovak Academy of Science, Dubravska cesta 9, 845 06 Bratislava, Slovakia; 7 Biomedical Sciences Research Center Al. Fleming, 16672 Vari, Greece; 8 Present Address: Department of Environmental and Natural Resources Management, University of Western Greece, 2 Seferi St, 30100 Agrinio, Greece

## Abstract

**Background:**

*Wolbachia* is a genus of endosymbiotic α-Proteobacteria infecting a wide range of arthropods and filarial nematodes. *Wolbachia* is able to induce reproductive abnormalities such as cytoplasmic incompatibility (CI), thelytokous parthenogenesis, feminization and male killing, thus affecting biology, ecology and evolution of its hosts. The bacterial group has prompted research regarding its potential for the control of agricultural and medical disease vectors, including *Glossina* spp., which transmits African trypanosomes, the causative agents of sleeping sickness in humans and nagana in animals.

**Results:**

In the present study, we employed a *Wolbachia* specific *16S rRNA* PCR assay to investigate the presence of *Wolbachia* in six different laboratory stocks as well as in natural populations of nine different *Glossina* species originating from 10 African countries. *Wolbachia* was prevalent in *Glossina morsitans morsitans*, *G. morsitans centralis* and *G. austeni* populations. It was also detected in *G. brevipalpis*, and, for the first time, in *G. pallidipes* and *G. palpalis gambiensis*. On the other hand, *Wolbachia* was not found in *G. p. palpalis*, *G. fuscipes fuscipes* and *G. tachinoides*. *Wolbachia* infections of different laboratory and natural populations of *Glossina* species were characterized using *16S rRNA*, the *wsp* (Wolbachia Surface Protein) gene and MLST (Multi Locus Sequence Typing) gene markers. This analysis led to the detection of horizontal gene transfer events, in which *Wobachia* genes were inserted into the tsetse flies fly nuclear genome.

**Conclusions:**

*Wolbachia* infections were detected in both laboratory and natural populations of several different *Glossina* species. The characterization of these *Wolbachia* strains promises to lead to a deeper insight in tsetse flies-*Wolbachia* interactions, which is essential for the development and use of *Wolbachia*-based biological control methods.

## Background

*Wolbachia* are a highly diverse group of intracellular, maternally inherited endosymbionts belonging to the α-Proteobacteria [1]. The bacteria infect a wide range of arthropods, including at least 65% of insect species [2-4], as well as filarial nematodes [5]. *Wolbachia* induce a range of reproductive abnormalities in their arthropod hosts, such as cytoplasmic incompatibility (CI), parthenogenesis, male-killing and feminization [1,6-11], while they have developed mutualistic associations with filarial nematodes [12-14]. The ability of *Wolbachia* to cause these reproductive phenotypes allows them to spread efficiently and rapidly into host populations [4,9]. *Wolbachia* has attracted much interest for its role in biological, ecological and evolutionary processes, as well as for its potential for the development of novel and environment friendly strategies for the control of insect pests and disease vectors [15-22].

Tsetse flies, the sole vectors of pathogenic trypanosomes in tropical Africa, infect many vertebrates, causing sleeping sickness in humans and nagana in animals [23]. It is estimated by the World Health Organization (WHO) that 60 million people in Africa are at risk of contracting sleeping sickness (about 40% of the continent's population). The loss of local livestock from nagana amounts to 4.5 billion U.S. dollars annually [24,25]. Thanks to a vigorous campaign led by the WHO and various NGOs, the infected population has declined to an estimated 10,000, following epidemics that killed thousands of Africans [26]. Given that the disease affects remote areas, it is, however, likely that many cases may remain unreported. Should active case finding and treatment be discontinued, it would be prudent to maintain vector surveillance and control measures to prevent (re)emergence of the disease as was witnessed in the early 1990’s in various parts of the continent [26,27].

*Wolbachia*-induced cytoplasmic incompatibility has been suggested as a potential tool to suppress agricultural pests and disease vectors [8,21,22,28-30]. Another potential control approach is based on a replacement strategy, where parasite-susceptible fly populations would be replaced with genetically modified strains that are unable to transmit the pathogenic parasites. Towards this end, a paratransgenic modification approach has been developed for tsetse flies. It has been possible to culture and genetically transform a tsetse flies symbiont, the commensal bacterium *Sodalis glossinidius*. The expression of biological anti-parasitic in *Sodalis* and reconstitution of tsetse flies with the recombinant symbionts can yield modified parasite resistant flies [31,32]. Methods that would drive the modified insects into natural population are, however, necessary to implement this approach. To this end, greater insight in tsetse flies-symbiont interactions, with focus on their implications for biological control methods, is essential [33].

The genus *Wolbachia* is highly diverse and is currently divided into 10 supergroups (A to K, although the validity of supergroup G is disputed) [34-40], while strain genotyping is most often based on a multi locus sequence typing system (MLST) which includes the sequences of five conserved genes (*gatB*, *coxA*, *hcpA*, *ftsZ* and *fbpA*), as well as on the amino acid sequences of the four hypervariable regions (HVRs) of the WSP protein [41]. Species of the genus *Glossina* (Diptera: Glossinidae) including *G. morsitans morsitans*, *G. austeni* and *G. brevipalpis* are known to harbour *Wolbachia* infections [42,43], which belong to supergroup A based on the *Wolbachia* surface protein (*wsp*) gene [42,44].

Several recent studies reported that *Wolbachia* genes, in some cases even large chromosomal segments, have been horizontally transferred to host chromosomes. Such events have been described in a variety of insect and nematode hosts, including the adzuki bean beetle *Callosobruchus chinensis*, the fruit fly *Drosophila ananassae*, a parasitoid wasp of the genus *Nasonia*, the mosquito *Aedes aegypti*, the pea aphid *Acyrthosiphon pisum*, the longicorn beetle *Monochamus alternatus* and filarial nematodes of the genera *Onchocerca*, *Brugia* and *Dirofilaria *[45-52]. Interestingly, some of these genes are highly transcribed suggesting that laterally transferred bacterial genes can be of functional importance [48-50].

In the present study, we report on the presence of *Wolbachia* infections in laboratory and natural populations of *Glossina* species. The characterization of these *Wolbachia* strains is based on the use of *16S rRNA*, *wsp* and MLST gene markers. In addition, we report horizontal gene transfer events of *Wolbachia* genes to *G. m. morsitans* chromosomes.

## Methods

### Sample collection and DNA isolation

*Glossina* specimens were collected in ten countries in Africa (Tanzania, South Africa, Zambia, Zimbabwe, Kenya, Senegal, Guinea, Ethiopia, Uganda, and Democratic Republic of Congo - Zaire). Upon their arrival in the lab, all tsetse flies specimens have been immediately used for DNA extraction. DNA samples were stored at -20^o^C until their use. Laboratory strains from FAO/IAEA (Seibersdorf), Yale University (EPH), Slovak Academy of Sciences (SAS-Bratislava), Kenya (KARI-TRC), Burkina Faso (CIRDES) and Antwerp were also included in the analysis. DNA from adult flies was isolated according to Abd-Alla et al. 2007 [53], using the Qiagen DNeasy kit (Qiagen, Valencia, CA), following the manufacturers’ instructions, except for the samples from Antwerp and Bratislava, to which the CTAB (Cetyl trimethylammonium bromide) DNA isolation method was applied [54]. *G. m. morsitans* fertile females were maintained on blood meals supplemented with 10% (w/v) yeast extract (Becton Dickinson) and 20 ug/ml of tetracycline. Flies were fed every 48h for the duration of their life span. The resulting progeny are aposymbiotic (Gmm^Apo^) in that they lack their natural endosymbionts, *Wigglesworthia* and *Wolbachia* (Alam and Aksoy, personal communication)*.* Aposymbiotic progeny were used for detection of nuclear *Wolbachia DNA*.

### PCR screen and MLST

A total of 3750 specimens of nine *Glossina* species (*G. m. morsitans*, *G. m. centralis*, *G. austeni*, *G. brevipalpis*, *G. pallidipes*, *G. p. palpalis*, *G. p. gambiensis*, *G. fuscipes fuscipes and G. tachinoides*) were screened for the presence of *Wolbachia* strains. The detection is based on the *Wolbachia 16S rRNA* gene and results in the amplification of an about 438 base pairs long DNA fragment with the *Wolbachia* specific primers wspecF and wspecR (see Additional file 1- Supplementary Table 1). The mitochondrial gene 12S rRNA was used as positive control for amplification; the primers 12SCFR (5'primer) 5'-GAG AGT GAC GGG CGA TAT GT-3’ and 12SCRR (3' primer) 5'-AAA CCA GGA TTA GAT ACC CTA TTA T-3' were used, which amplify a 377 bp fragment of the gene [55]. PCR amplifications were performed in 20 μl reaction mixtures containing 4 μl 5x reaction buffer (Promega), 1.6 μl MgCl_2_ (25mM), 0.1 μl deoxynucleotide triphosphate mixture (25 mM each), 0.5 μl of each primer (25 μM), 0.1 μl of *Taq* (Promega 1U/μl), 12.2 μl water and 1 μl of template DNA. The PCR protocol was: 35 cycles of 30 sec at 95°C, 30 sec at 54°C and 1 min at 72 °C.

The *Wolbachia* strains present in eleven selected *Wolbachia*-infected *Glossina* specimens from different areas and species were genotyped with MLST- and *wsp*-based approaches. The *wsp* and MLST genes (*gatB*, *coxA*, *hcpA*, *fbpA* and f*tsZ*) were amplified using the respective primers reported in [41] (see Additional file 1- Supplementary Table 1). Gene fragments were amplified using the following PCR mixes: 4 μl of 5x reaction buffer (Promega), 1.6 μl MgCl_2_ (25mM), 0.1 μl deoxynucleotide triphosphate mixture (25 mM each), 0.5 μl of each primer (25 μM), 0.1 μl of *Taq* (Promega 1U/μl), 12.2 μl water and 1 μl of template. PCR reactions were performed using the following program: 5 min of denaturation at 95 °C, followed by 35 cycles of 30 sec at 95°C, 30 sec at the appropriate temperature for each primer pair (52°C for *ftsZ*, 54°C for *gatB*, 55°C for *coxA*, 56°C for *hcpA*, 58°C for *fbpA* and *wsp*) and 1 min at 72 °C. All reactions were followed by a final extension step of 10 min at 72°C.

Given the presence of products of unpredicted size, all PCR products of genes *16S rRNA*, *wsp* and MLST from the eleven selected populations were ligated into a vector (pGEM-T Easy Vector System) according to the manufacturer’s instructions and then transformed into competent DH5α cells, which were plated on ampicillin/X-gal selection plates (the exception being *G. m. centralis*, for which direct sequencing of PCR products was employed) Three to six clones were directly subjected to PCR using the primers T7 and SP6. For each sample, a majority-rule consensus sequence was created. The colony PCR products were purified using a PEG (Polyethylene glycol) - NaCl method [56]. Both strands of the products were sequenced using the universal primers T7 and SP6. A dye terminator-labelled cycle sequencing reaction was conducted with the BigDye Terminator v3.1 Cycle Sequencing Kit (PE Applied Biosystems). Reaction products were analysed using an ABI PRISM 310 Genetic Analyzer (PE Applied Biosystems).

### Tissue specific detection of cytoplasmic and nuclear *Wolbachia* DNA

To detect the presence of cytoplasmic or nuclear *Wolbachia* genes in different tissues, DNA extracts were prepared from gut, ovary, testes, and carcasses (remaining fly tissues after organ extraction) of *Wolbachia* infected and tetracycline-treated (*Wolbachia*-free) teneral two-day old *G. m. morsitans* female and male adult flies from the Yale University laboratory colony. Dissections were performed in 1X PBST ((3.2 mM Na2HPO4, 0.5 mM KH2PO4, 1.3 mM KCl, 135 mM NaCl, 0.05% Tween 20, pH 7.4), and dissected tissues were placed in 200 μl of lysis buffer (Qiagen, Valencia, CA). The DNA was isolated using a Qiagen DNeasy kit (Qiagen, Valencia, CA) following the manufacturer’s instructions. PCR amplication of *16S rRNA*, *fbpA*, and *wsp* were performed using the primers wspecF/wspecR, fbpA_F1 / fbpA_R1 and 81F / 691R, respectively [2,41,57] (see Additional file 1- Supplementary Table 1). PCR mixes of 25 μl contained 5 μl of 5x reaction buffer (Promega, Madison, WI), 3 μl MgCl_2_ (25mM), 0.5 μl deoxynucleotide triphosphate mixture (25 mM each), 0.5 μl of each primer (10 μM), 0.125 μl of Taq (Promega, Valencia, CA) (1U/μl), 14.375 μl water and 1 μl of template DNA. The PCR protocol was: 35 cycles of 30 sec at 95°C, 30 sec at 54°C and 1 min at 72 °C.

### Phylogenetic analysis

All *Wolbachia* gene sequences generated in this study were manually edited with SeqManII by DNAStar and aligned using MUSCLE [58] and ClustalW [59], as implemented in Geneious 5.3.4 [60], and adjusted by eye. Phylogenetic analyses were performed using Bayesian Inference (BI) and Maximum-Likelihood (ML) estimation for a concatenated data set of the protein-coding genes (*gatB*, *fbpA*, *hcpA*, *ftsZ* and *coxA*) and for *wsp* separately. For the Bayesian inference of phylogeny, PAUP version 4.0b10 [61] was used to select the optimal evolution model by critically evaluating the selected parameters using the Akaike Information Criterion [62]. For the concatenated data and the *wsp* set, the submodel GTR+I+G was selected. Bayesian analyses were performed as implemented in MrBayes 3.1 [63]. Analyses were initiated from random starting trees. Four separate runs, each composed of four chains, were run for 6,000,000 generations. The cold chain was sampled every 100 generations, and the first 20,000 generations were discarded. Posterior probabilities were computed for the remaining trees. ML trees were constructed using MEGA 5.0 [64], with gamma distributed rates with 1000 bootstrap replications, and the method of Jukes and Cantor [65] as genetic distance model.

**Nucleotide sequence accession numbers**. All MLST, *wsp* and *16S rRNA* gene sequences generated in this study have been deposited into GenBank under accession numbers JF494842 to JF494922 and JF906102 to JF906107.

## Results

### *Wolbachia* infection prevalence in different populations

The presence of *Wolbachia* was investigated in nine species within the three subgenera of *Glossina*. A total of 551 laboratory and 3199 field-collected adult flies, originating from 10 African countries, were tested using a *Wolbachia* specific *16S rRNA*-based PCR assay (Table 1). The prevalence of *Wolbachia* infections differed significantly between the various populations of *Glossina* (Table 1). *Wolbachia* infections were detected in multiple species of the *morsitans* complex: *G. m. morsitans*, *G. m. centralis*, *G. pallidipes* and *G. austeni*, in the *fusca* complex in *G. brevipalpis*, while it was absent in the analysed species from the *palpalis* complex: *G. p. palpalis*, *G. fuscipes* and *G. tachinoides*. *Wolbachia* was also detected in just two out of 644 individuals of *G. p. gambiensis*.

**Table 1:** *Wolbachia* prevalence in laboratory lines and natural populations of different *Glossina* species.

*Glossina* species	Country (area, collection date)	Prevalence
*G. m. morsitans*	Zambia (MFWE, Eastern Zambia, 2007)	(122/122) 100.0%
	KARI-TRC lab-colony (2008)^1^	(89/89) 100.0%
	Tanzania (Ruma, 2005)	(100/100) 100.0%
	Zimbabwe (Gokwe, 2006)	(7/74) 9.5%
	Zimbabwe (Kemukura, 2006)	(26/26) 100.0%
	Zimbabwe (M.Chiuy, 1994)	(33/36) 91.7%
	Zimbabwe (Makuti, 2006)	(95/99) 96.0%
	Zimbabwe (Mukond, 1994)	(35/36) 97.2%
	Zimbabwe (Mushumb, 2006)	(3/8) 37.5%
	Zimbabwe (Rukomeshi, 2006)	(98/100) 98.0%
	Yale lab-colony (2008)^2^	(5/5) 100.0%
	Antwerp lab-colony (2010)^3^	(10/10) 100.0%
	Bratislava lab-colony (2010)^4^	(5/5) 100.0%

*G. pallidipes*	Zambia (MFWE, Eastern Zambia, 2007)	(5/203) 2.5%
	KARI-TRC lab-colony (2008)	(3/99) 3.0%
	Kenya (Mewa, Katotoi and Meru national park, 2007)	(0/470) 0.0%
	Ethiopia (Arba Minch, 2007)	(2/454) 0.4%
	Seibersdorf lab-colony (2008)^5^	(0/138) 0.0%
	Tanzania (Ruma, 2005)	(3/83) 3.6%
	Tanzania (Mlembuli and Tunguli, 2009)	(0/94) 0.0%
	Zimbabwe (Mushumb, 2006)	(0/50) 0.0%
	Zimbabwe (Gokwe, 2006)	(0/150) 0.0%
	Zimbabwe (Rukomeshi, 2006)	(5/59) 8.5%
	Zimbabwe (Makuti, 2006)	(4/96) 4.2%

*G. austeni*	Tanzania (Jozani, 1997)	(22/42) 52.4%
	Tanzania (Zanzibar, 1995)	(75/78) 96.2%
	South Africa (Zululand, 1999)	(79/83) 95.2%
	Kenya (Shimba Hills, 2010)	(30/30) 100.0%

*G. p. palpalis*	Seibersdorf lab-colony (1995)^6^	(0/36) 0.0%
	Democratic Republic of Congo (Zaire, 1995)	(0/48) 0.0%

*G. p. gambiensis*	CIRDES lab-colony (1995)^7^	(0/32) 0.0%
	CIRDES lab-colony (2005; this colony is now also established at Seibersdorf)^7^	(0/57) 0.0%
	Senegal (Diacksao Peul and Pout, 2009)	(1/188) 0.5%
	Guinea (Kansaba, Mini Pontda, Kindoya and Ghada Oundou, 2009)	(0/180) 0.0%
	Guinea (Alahine, 2009)	(0/29) 0.0%
	Guinea (Boureya Kolonko, 2009)	(0/36) 0.0%
	Guinea (Fefe, 2009)	(0/29) 0.0%
	Guinea (Kansaba, 2009)	(0/19) 0.0%
	Guinea (Kindoya, 2009)	(1/12) 8.3%
	Guinea (Lemonako, 2009)	(0/30) 0.0%
	Guinea (Togoue, 2009)	(0/32) 0.0%

*G. brevipalpis*	Seibersdorf lab-colony (1995)^8^	(14/34) 41.2%
	South Africa (Zululand, 1995)	(1/50) 2.0%

*G. f. fuscipes*	Seibersdorf lab-colony (1995)^9^	(0/36) 0.0%
	Uganda (Buvuma island, 1994)	(0/53) 0.0%

*G. m. centralis*	Yale lab-colony (2008; this colony no longer exists at Yale)^10^	(3/3) 100.0%

*G. tachinoides*	Seibersdorf lab-colony (1995; this colony no longer exists at Seibersdorf)^11^	(0/7) 0.0%

Numbers in parentheses indicate the *Wolbachia* positive individuals/total individuals analyzed from each population.

^1^KARI-TRC is located in Nairobi, Kenya and its laboratory colony was established through Bristol lab (the start-up flies of Bristol lab were collected in Zimbabwe).

^2^The Yale lab-colony was also established through Bristol lab.

^3^The Antwerp lab-colony was established in its present form in 1993. Its start-up flies were originally collected in Kariba (Zimbabwe) in 1967 and Handemi (Tanzania) in 1973 which were pooled in 1978 after a series of enrichments from flies of Bristol, University of Alberta (Canada) and IAEA lab-colonies.

^4^The Bratislava lab-colony was established from a colony in Seibersdorf, which itself came from Zimbabwe via Bristol (same as ^2^ above).

^5^The Seibersdorf lab-colony start-up flies were collected in Tororo, Uganda in 1975.

^6^The Seibersdorf lab-colony start-up flies were collected in Nigeria. This colony was transferred to CIRAD, Montpellier, France in 2009.

^7^The CIRDES lab-colony start-up flies were collected in Burkina-Faso in early 1990s.

^8^The Seibersdorf lab-colony start-up flies were collected in Shimba Hills, Kenya. This colony was transferred to Onderstepoort, South Africa in 2009.

^9^The Seibersdorf lab-colony was established from Central African Republic in 1986. This colony was transferred to Bratislava, Slovakia in 2009.

^10^The Yale lab-colony was established through Bristol lab.

^11^The Seibersdorf lab-colony was established through CIRDES lab, which still has the colony.

Despite the heterogenous infections found in field populations, *Wolbachia* infection was fixed in the laboratory colonies of *G. m. morsitans*, and *G. m. centralis*. On the other hand, the infection was not fixed in laboratory colonies of *G. brevipalpis* and *G. pallidipes* and was completely absent from the laboratory colonies of the *palpalis* group species: *G. p. palpalis*, *G. p. gambiensis*, *G. f. fuscipes* and *G. tachinoides*.

*Wolbachia* prevalence ranged from 9.5 to 100% in natural populations of *G. m. morsitans*, from 52 to 100% in *G. austeni*, while it was only 2% in *G. brevipalpis*. Interestingly, previous studies on *G. pallidipes* and *G. p. gambiensis* natural populations did not observe any *Wolbachia* infection in these species. Our study did not find any evidence for *Wolbachia* infections in the screened natural populations of *G. p. palpalis* and *G. f. fuscipes*.

It is also interesting to note that the prevalence of *Wolbachia* infection was not homogenous and varied in different geographic populations for the same species. For example, the infection was fixed in natural populations of *G. m. morsitans* in Zambia and Tanzania while in Zimbabwe, two different sites exhibited 9.5% (Gokwe) and 100% (Kemukura) prevalence respectively.

### Genotyping tsetse flies *Wolbachia* strains

The bacterial strains present in each of the eleven *Wolbachia*-infected *Glossina* populations (seven natural and four laboratory), representing six species, were genotyped using MLST analysis (Table 2). A total of nine allelic profiles or Sequence Types (ST) was found in tsetse flies *Wolbachia* strains. All of them were new STs, based on the available data in the *Wolbachia* MLST database. The STs of the *Wolbachia* strains infecting the laboratory population of *G. m. centralis* and two out of the four natural populations of *G. m. morsitans* (12.3A, 32.3D) were identical. All *Wolbachia* strains infecting *G. m. morsitans* (except 24.4A) and *G. m. centralis* populations belong to the same sequencing complex, since they share at least three alleles. The MLST analysis showed the presence of seven *gatB*, seven *coxA*, four *hcpA*, seven *ftsZ* and four *fbpA* alleles. This analysis also revealed the presence of new alleles for all loci: five for *gatB*, four for *coxA*, two for *hcpA*, five for *ftsZ* and two for *fbpA* (Table 2).

**Table 2:** *Wolbachia* MLST allelic profiles for 11 populations of *Glossina*

Code	Species	Country (area, collection date)	*Wolbachia* MLST					
	
			ST	*gatB*	*coxA*	*hcpA*	*ftsZ*	*fbpA*
12.3A	*G. m. morsitans*	Zambia (MFWE, Eastern Zambia, 2007)	**226**	141	127	23	114	15
32.3D	*G. m. morsitans*	Zimbabwe (Makuti, 2006)	**226**	141	127	23	114	15
GmcY	*G. m. centralis*	Yale lab-colony (2008)	**226**	141	127	23	114	15
30.9D	*G. m. morsitans*	Zimbabwe (Rukomeshi, 2006)	**227**	141	127	23	115	15
GmmY	*G. m. morsitans*	Yale lab-colony (2008)	**228**	8	127	23	113	15
24.4A	*G. m. morsitans*	KARI-TRC lab-colony (2008)	**229**	142	128	23	113	15
09.7G	*G. brevipalpis*	Seibersdorf lab-colony (1995)	**230**	143	129	23	56	15
05.2B	*G. austeni*	South Africa (Zululand, 1999)	**231**	128	109	127	98	20
GauK	*G. austeni*	Kenya (Shimba Hills, 2010)	**197**	128	108	127	98	20
15.5B	*G. pallidipes*	Ethiopia (Arba Minch, 2007)	**232**	144	47	149	116	202
405.11F	*G. p. gambiensis*	Guinea (Kindoya, 2009)	**233**	145	130	150	117	203

Identical nucleotide sequences at a given locus for different strain were assigned the same arbitrary allele number. Each strain was then identified by the combination of the five MLST allelic numbers, representing its allelic profile. Each unique allelic profile was assigned an ST (Sequence Type), which ultimately characterizes a strain [41].

The same eleven samples were also genotyped using the *wsp* gene: nine alleles were detected. For all tsetse flies *Wolbachia* strains, the WSP HVR profile, a combination of the four HVR amino acid haplotypes, was determined as described previously [41] (Table 3). A total of eight WSP HVR profiles were identified; six of them were new in the *Wolbachia* WSP database. The WSP HVR profile of the *Wolbachia* strains infecting (a) the natural population (12.3A) and the Yale lab colony (GmmY) of *G. m. morsitans*, (b) two natural populations of *G. m. morsitans* (32.3D and 30.9D) and (c) two natural populations of *G. austeni* (GauK and 05.2B) were identical. On the other hand, the *Wolbachia* strains infecting the KARI lab colony of *G. m. morsitans* (24.4A) as well as *G. m. centralis* (GmcY), *G. pallidipes* (15.5B), *G. brevipalpis* (09.7G) and *G. p. gambiensis* (405.11F) had unique WSP profiles. It is also interesting to note that three *Wolbachia* strains infecting *G. m. morsitans* (32.3D, 30.9D) and *G. brevipalpis* (09.7G) shared three HVR haplotypes (HVR2-4). Another triplet of strains infecting *G. m. morsitans* (32.3D, 30.9D and 24.4A) also shared three HVR haplotypes (HVR1, 2 and 4). The overall number of unique haplotypes per HVR varied. The WSP profile analysis showed the presence of seven HVR1, four HVR2, six HVR3 and five HVR4 haplotypes. The analysis also revealed the presence of new haplotypes: four for HVR1, two for HVR2, four HVR3 and one for HVR4 (Table 3).

**Table 3:** *Wolbachia* WSP HVR profiles for 11 populations of *Glossina*

Code	Species	Country (area, collection date)	***wsp***	HVR1	HVR2	HVR3	HVR4
12.3A	*G. m. morsitans*	Zambia (MFWE, Eastern Zambia, 2007)	**548**	192	9	12	202
32.3D	*G. m. morsitans*	Zimbabwe (Makuti, 2006)	**356**	142	9	12	9
GmcY	*G. m. centralis*	Yale lab-colony (2008)	**550**	193	9	221	202
30.9D	*G. m. morsitans*	Zimbabwe (Rukomeshi, 2006)	**356**	142	9	12	9
GmmY	*G. m. morsitans*	Yale lab-colony (2008)	**548**	192	9	12	202
24.4A	*G. m. morsitans*	KARI-TRC lab-colony (2008)	**549**	142	9	223	9
09.7G	*G. brevipalpis*	Seibersdorf lab-colony (1995)	**11**	9	9	12	9
05.2B	*G. austeni*	South Africa (Zululand, 1999)	**551**	180	40	210	18
GauK	*G. austeni*	Kenya (Shimba Hills, 2010)	**507**	180	40	210	18
15.5B	*G. pallidipes*	Ethiopia (Arba Minch, 2007)	**552**	195	224	224	63
405.11F	*G. p. gambiensis*	Guinea (Kindoya, 2009)	**553**	194	223	222	220

WSP profiles of *Wolbachia* for 11 populations of *Glossina*, defined as the combination of the four HVR amino acid haplotypes. Each WSP amino acid sequence (corresponding to residues 52 to 222 of the wMel sequences) was partitioned into four consecutive sections, whose breakpoints fall within conserved regions between the hypervariable regions, as follows: HVR1 (amino acids 52 to 84), HVR2 (amino acids 85 to 134), HVR3 (amino acids 135 to 185), and HVR4 (amino acids 186 to 222) [41].

### Phylogenetic analysis

**Figure 1:**
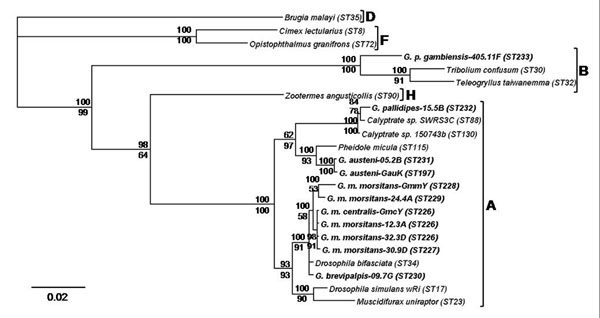
**Bayesian inference phylogeny based on the concatenated MLST data (2,079 bp).** The topology resulting from the Maximum Likelihood method was similar. The 11 *Wolbachia* strains present in *Glossina* are indicated in bold letters, and the other strains represent supergroups A, B, D, F and H. Strains are characterized by the names of their host species and ST number from the MLST database. *Wolbachia* supergroups are shown to the right of the host species names. Bayesian posterior probabilities (top numbers) and ML bootstrap values based on 1000 replicates (bottom numbers) are given (only values >50% are indicated).

**Figure 2:**
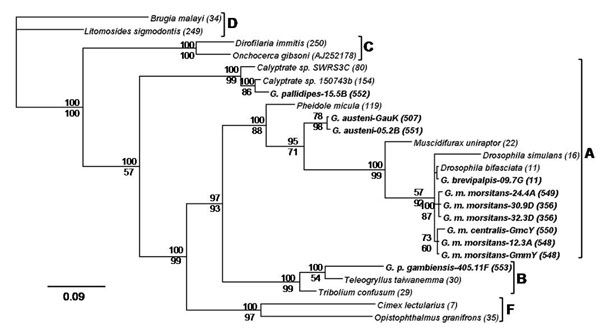
**Bayesian inference phylogeny based on the *wsp* sequence.** The topology resulting from the Maximum Likelihood method was similar. The 11 *Wolbachia* strains present in *Glossina* are indicated in bold letters, and the other strains represent supergroups A, B, C, D and F. Strains are characterized by the names of their host species and their *wsp* allele number from the MLST database (except *O. gibsoni* for which the GenBank accession number is given). *Wolbachia* supergroups are shown to the right of the host species names. Bayesian posterior probabilities (top numbers) and ML bootstrap values based on 1000 replicates (bottom numbers) are given (only values >50% are indicated).

Phylogenetic analysis based on a concatenated dataset of all MLST loci revealed that the *Wolbachia* strains infecting *G. m. morsitans*, *G. m. centralis*, *G. brevipalpis*, *G. pallidipes* and *G. austeni* belong to supergroup A, while the *Wolbachia* strain infecting *G. p. gambiensis* fell into supergroup B (Fig. 1). The respective phylogenetic analysis based on the *wsp* gene dataset confirmed these results (Fig. 2). Phylogenetic reconstructions for concatenated alignments of MLST loci and *wsp* sequences showed similar results by both Bayesian inference and Maximum Likelihood methods. The Bayesian phylogenetic trees are presented in Figures 1 and 2 while the Maximum Likelihood trees are shown in Supplementary Figures 1 and 2 (Additional Files 2 and 3). The tsetse flies *Wolbachia* strains within the supergroup A form three different clusters. The first cluster includes the *Wolbachia* strains present in *G. m. morsitans*, *G. m. centralis* and *G. brevipalpis*. This cluster is closely related to *Wolbachia* strains infecting the fruit fly *Drosophila bifasciata*. The second cluster includes the *Wolbachia* strains infecting *G. austeni* populations and is distantly related to the strain present in *Pheidole micula*. The third cluster contains only the *Wolbachia* strain present in *G. pallidipes* and is closely related to *Wolbachia* strains present in Dipteran host species. The B-supergroup *Wolbachia* strain infecting *G. p. gambiensis* clusters with strains present in *Tribolium confusum* and *Teleogryllus taiwanemma* (Figs 1 and 2).

### Horizontal transfer of *Wolbachia* genes to the *G. m. morsitans* genome

**Figure 3:**
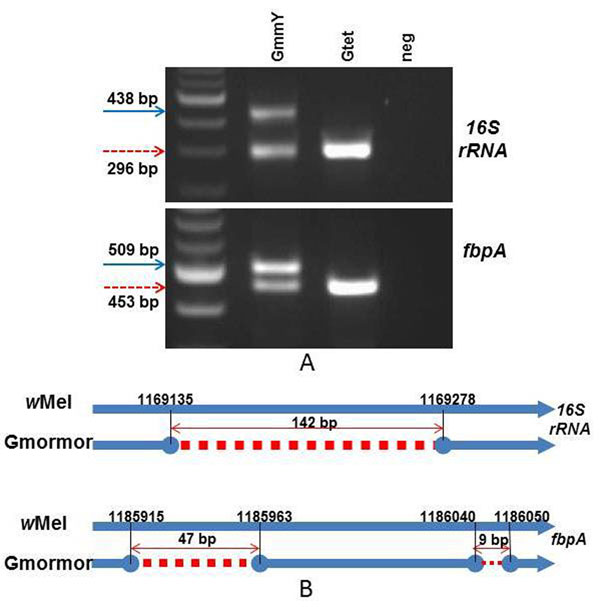
**Overview of deleted fragments in two *Wolbachia* genes** A) PCR amplified products from *G. m. morsitans* (GmmY and Gtet) of the *16S rRNA* and *fbpA* genes were resolved on 2.5% agarose gels stained with ethidium bromide. A 100-bp ladder was used as size standard. The input of the negative (neg) control was water. B) *16S rRNA* and *fbpA* fragments from tsetse flies Wolbachia strains aligned with the corresponding regions of strain *w*Mel. Red dashes represent the deletion region, the numbers show the positions before and after the deletions in respect to the *w*Mel genome. The blue arrows represent the corresponding *w*Mel genes. Deleted fragments were detected in *G. m. morsitans* samples (Gmormor: GmmY, 12.3A, 24.4A, 30.9D, 32.3D and Gtet). The right-left red arrows below the number indicate the size of deletion in base pairs.

During the *Wolbachia*-specific *16S rRNA*-based PCR screening of laboratory and natural *G. m. morsitans* populations, the presence of two distinct PCR amplification products was observed: one compatible with the expected size of 438 bp and a second smaller product of about 300 bp (Fig. 3a). Both PCR products were sequenced and confirmed to be of *Wolbachia* origin. The 438 bp product corresponded to the expected *16S rRNA* gene fragment, while the shorter product contained a deletion of 142 bp (Fig. 3b). The 296 bp shorter version of the *16S rRNA* gene was detected in all five individuals analyzed from *G. m. morsitans* colony individuals, as well as in DNA prepared from the tetracycline-treated (*Wolbachia*-free) *G. m. morsitans* samples, suggesting that it is of nuclear, and not cytoplasmic origin. This finding implies that the *16S rRNA* gene segment was most likely transferred from the cytoplasmic *Wolbachia* to the *G. m. morsitans* genome, where it was pseudogenized through a deletion event. During the MLST analysis of the *Wolbachia* strain infecting *G. m. morsitans*, a similar phenomenon was observed for gene *fbpA*. PCR analysis showed the presence of two distict amplicons (Fig. 3a). Sequence analysis revealed that the larger 509 bp fragment was of the expected size, while the smaller fragment (453 bp in size) contained two deletions of 47 bp and 9 bp, respectively (Fig. 3b). The *Wolbachia*-free *G. m. morsitans* line contained only the smaller 453 bp version of the *fbpA* gene, suggesting again that this gene fragment is the result of a horizontal gene transfer event to the host chromosome.

### Tissue specific detection of cytoplasmic and nuclear *Wolbachia* markers

**Figure 4:**
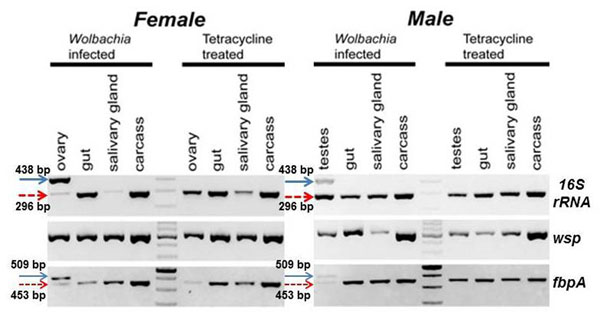
**Tissue tropism of *Wolbachia* infections in *G. m. morsitans***. *G. m. morsitans* (GmmY and Gtet) PCR amplicons of genes *16S rRNA*, *wsp*, and *fbpA* were resolved on a 2% agarose gel stained by ethidium bromide. The arrow with the solid line represents the cytoplasmic *Wolbachia* PCR product restricted to the reproductive tissues, and the arrow with the dashed line represents the PCR product found in all tissues tested. A 100 bp DNA ladder is used as size marker

The tissue specific distribution of the *Wolbachia* markers in *G. m. morsitans* were tested in ovary, salivary gland, midgut and carcass in normal and tetracycline-treated (*Wolbachia*-cured) flies. Two *16S rRNA* PCR products (438 and 296 bp as described in Figure 3, corresponding to cytoplasmic and nuclear *Wolbachia* markers) could be amplified from ovary and testes tissues of uncured flies, while only the truncated 296 bp product that corresponds to the nuclear *Wolbachia* marker was amplified from all of the tissues (Figure 4). In contrast, the fragment that corresponds to the cytoplasmic 16S rRNA marker could not be amplified from any of the tissues of *Wolbachia* cured tetracycline-treated flies, including the reproductive organs (ovary and testes) (Fig. 4). The amplification of the larger product that corresponds to the cytoplasmic *Wolbachia* only from testes and ovary tissues of adults suggests that *Wolbachia* is restricted to the gonadal tissues in this species. Unlike for the 16S *rRNA*, a single *wsp* PCR product was observed in all tissues of *Wolbachia* infected and cured adults (Fig. 4). While it was not possible to differentiate between amplifications of cytoplasmic and nuclear *Wolbachia*, amplification from tetracycline treated adults suggests a horizontal transfer event also for the *wsp* gene. The size heterogeneity was also observed for *fbpA*. The larger 509 bp amplification which corresponds to the cytoplasmic marker was restricted to the reproductive tissues of the tsetse flies while the smaller derived 453 bp product corresponding to the nuclear marker was present in all tissues of infected and cured adults, suggesting horizontal transfer of *fbpA* to the *G. m. morsitans* genome (Fig. 4).

## Discussion

### Prevalence of *Wolbachia* in *Glossina* species

Our study suggests that *Wolbachia* infections are present in multiple species of the genus *Glossina*; however, the prevalence of infections in laboratory colonies versus natural populations and the *Wolbachia* strain harboured in the different species varies. The infection seems to be prevalent to the *morsitans* (savannah) group, which includes the species *G. m. morsitans*, *G. m. centralis* and *G. austeni*. In addition, uncured laboratory colonies largely show fixation, suggestive of active cytoplasmic incompatibility (Alam and Aksoy, personal communication). *Wolbachia* was also detected in the fusca (forest) group, which includes *G. brevipalpis*. In contrast, *Wolbachia* infection seems to be largely absent from the *palpalis* (riverine) group, which includes *G. f. fuscipes*, *G. tachinoides* and *G. p. palpalis*. It should be mentioned, however, that our results depend on the PCR-amplification conditions employed in this study and the presence of low density *Wolbachia* infections in these species, as has been reported for other insect species [66-68], cannot be excluded. Given that our screen was based on specimens collected during 1994-2010 (see Table 1), new screens should provide information on the dynamics of infection and the expression of cytoplasmic incompatibility.

The abovementioned data are in accordance with previous reports that detected *Wolbachia* in *G. m. morsitans*, *G. m. centralis*, *G. brevipalpis* and *G. austeni *[42,43]. For the first time our study reports the presence of *Wolbachia*, albeit at very low prevalence, in *G. pallidipes* (*morsitans* group) and in *G. p. gambiensis* (palpalis group). The infection was only detected in 22 out of 1896 *G. pallidipes* and in 2 out of 644 *G. p. gambiensis* individuals; in both species, the infection was present in different populations, as shown in Table 1. Whether the presence of *Wolbachia* in these two species is a result of horizontal transfer, hybrid introgression or co-divergence in the *morsitans* and *palpalis* species complexes, as has recently been shown in other species complexes, has to await investigation [69-71].

The prevalence of *Wolbachia* was not homogenous among the different natural populations of *G. m. morsitans*. For example, in the area Gokwe (Zimbabwe), the infection prevalence was almost nine times lower than the average of the other areas. *Glossina* populations have been shown to exhibit extensive genetic structuring; of which the observed *Wolbachia* infection dynamics may be a result [72,73]. Similar observations were made in *G. austeni* natural populations, where the *Wolbachia* infection was 98% in a South African population while the infection was 48% in a Kenyan population sampled in 1998 [42]. These data suggest that geography may influence *Wolbachia* prevalence as reported previously for field populations of spider *Hylyphantes graminicola *[74]. Further research on the heterogeneous distribution of *Wolbachia* infection in field populations could shed more light on the functional role of this endosymbiont in tsetse flies biology, ecology and evolution.

### Genotyping - phylogeny

The MLST- and *wsp*-based sequence analysis indicates that all but one of the *Wolbachia* strains infecting *Glossina* species belong to supergroup A; the exception being the bacterial strain infecting *G. p. gambiensis*, which belongs to supergroup B. The supergroup A tsetse flies *Wolbachia* strains are members of three separate and distantly related groups. Our results are in accordance with two previous studies that relied on just the *wsp* phylogeny but indicated a similar topology [42,44]. The phylogenetic analyses strongly suggest the presence of distantly related *Wolbachia* strains in tsetse flies species and support the hypothesis that horizontal transmission of *Wolbachia* between insect species from unrelated taxa has extensively occurred, as has been reported in the spider genus *Agelenopsis *[70], in the wasp genus *Nasonia *[71], in the acari genus *Bryobia *[40] and in the termites of genus *Odontotermes *[75]. On the other hand, the sibling species *G. m. morsitans* and *G. m. centralis* carry closely related *Wolbachia* strains, which have identical ST and differ only in the sequence of the fast evolving *wsp* gene, which suggests host-symbiont co-divergence. In addition, field populations of *G. m. morsitans* from different locations of Africa harbor very closely related *Wolbachia* strains, suggesting that the geographical origin of their hosts did not impact significantly *Wolbachia* strain divergence. Our findings are in agreement with reports on dipteran hosts associated with mushrooms [76] and on the spider Hylyphantes graminicola [74]. On the other hand, studies on fig wasps [77] and ants [78] showed considerable association between biogeography and strain similarity.

### Horizontal gene transfer

The evolutionary fate of any host-bacterial symbiotic association depends on the modes of transmission of the bacterial partner, vertical, horizontal or both. Additionally, horizontal gene (or genome) transfer events may also be important. Our data suggest that at least three genes (*16S rRNA*, *fbpA* and *wsp*) of the *Wolbachia* strain infecting *G. m. morsitans* have been transferred to the host genome (Figures 3 and 4). This transfer is supported by the amplification of derivative copies of *fbpA* and *16S rRNA*, and of *wsp* in tissues from tetracycline-treated *G. m. morsitans* (Figure 4). The results suggest that *fbpA* and *16S rRNA* have been pseudogenized through the accumulation of deletions, consistent with previous studies [45,46,51]. The transfer events were detected both in laboratory and natural populations, suggesting that they are the result of the long co-evolution of the host-*Wolbachia* associations. Interestingly, neither cytoplasmic *Wolbachia* infections nor chromosomal insertions were detected in the sibling species *G. m. centralis*, suggesting that the horizontal transfer event took place after the divergence of these two species. Our preliminary and ongoing studies indicate that chromosomal insertions with *Wolbachia* sequences may be more extensive than reported here (Aksoy and Bourtzis, unpublished observations). Similar horizontal transfer events have been reported for other *Wolbachia*-infected hosts [45-52]. It is worth noting that in some cases, horizontally transferred *Wolbachia* genes are expressed from the host genome, as reported in the mosquito *Aedes aegypti* and in the pea aphid *Acyrthosiphon pisum*, where the *Wolbachia*-like genes are expressed in salivary glands and in the bacteriocyte, respectively [48-50]. The release of the *G. morsitans morsitans* genome will allow us to further examine, by both *in silico* and molecular analysis, the extent of the horizontal gene transfer of the *Wolbachia* sequences into the tsetse fly nuclear genome and whether these genes are expressed.

## Conclusions

*Wolbachia* is present in both laboratory and natural populations of *Glossina* species. Tsetse flies *Wolbachia* strains were characterized based on *16S rRNA*, *wsp* and MLST gene markers. In addition, horizontal gene transfer events of *Wolbachia* genes into tsetse fly chromosomes were detected and characterized. The detailed characterization of *Wolbachia* infections is a crucial step towards an adequate understanding of tsetse flies-*Wolbachia* interactions, which is essential for the development and implementation of *Wolbachia*-based biological control approaches.

## Authors' contributions

Conceived and designed experiments: Abd-Alla Adly, Serap Aksoy, Kostas Bourtzis

Experimental work: Vangelis Doudoumis, George Tsiamis, Florence Wamwiri, Corey Brelsfoard, Uzma Alam, Emre Aksoy, Stelios Dalaperas, Abd-Alla Adly

Data analysis: Vangelis Doudoumis, George Tsiamis, Corey Brelsfoard, Aksoy Serap, Kostas Bourtzis

Contributed reagents/materials/analysis tools: Johnson Ouma, Peter Takac

Manuscript writing and editing: Vangelis Doudoumis, George Tsiamis, Corey Brelsfoard, Abd-Alla Adly, Aksoy Serap, Kostas Bourtzis

## Competing interests

The authors declare that they have no competing interests.

## Supplementary Material

Additional file 1 Supplementary Table 1: Primers used in the present study. Click here for file

Additional file 2 **Supplementary Figure 1: Maximum likelihood inference phylogeny based on the concatenated MLST data, 2,079 bp.** (Please note that tree has been rooted to the supergroup D sequences). Click here for file

Additional file 3 **Supplementary Figure 2: Maximum likelihood inference phylogeny based on the on the *wsp* sequence.** (Please note that tree has been rooted to the supergroup D sequences). Click here for file
