# Supplementary material for: Detection and characterization of Wolbachia infections in laboratory and natural populations of different species of tsetse flies (genus Glossina)
Source: BMC Microbiol. 2012 Jan 18;12(Suppl 1):S3. doi: 10.1186/1471-2180-12-S1-S3 (PMC3287514; doi:10.1186/1471-2180-12-S1-S3)
Supplement: Additional file 1 — Supplementary Table 1: Primers used in the present study. [file 1471-2180-12-S1-S3-S1.doc]

**Additional files**

**Additional file 1.**Supplementary Table 1. PCR primers used

| Locus code  (*w*Mel) | Gene | Product | Name | Primer sequence 5’- 3’ | Gene length  (bp)^a^ | MLST fragment  size (bp) | References |
| --- | --- | --- | --- | --- | --- | --- | --- |
| WD_0146 | *gatB* | glutamyl-tRNA(Gln) amidotransferase, subunit B | gatB_F1  gatB_R1 | GAKTTAAAYCGYGCAGGBGTT  TGGYAAYTCRGGYAAAGATGA | 1,425 | 369 | Baldo et al. 2006 [41] |
| WD_0301 | *coxA* | cytochrome c oxidase, subunit I | coxA_F1  coxA_R1 | TTGGRGCRATYAACTTTATAG  CTAAAGACTTTKACRCCAGT | 1,551 | 402 | Baldo et al. 2006 [41] |
| WD_0484 | *hcpA* | conserved hypothetical protein | hcpA_F1  hcpA_R1 | GAAATARCAGTTGCTGCAAA  GAAAGTYRAGCAAGYTCTG | 741 | 444 | Baldo et al. 2006 [41] |
| WD_0723 | *ftsZ* | cell division protein | ftsZ_F1  ftsZ_R1 | ATYATGGARCATATAAARGATAG  TCRAGYAATGGATTRGATAT | 1,197 | 435 | Baldo et al. 2006 [41] |
| WD_1238 | *fbpA* | fructose-bisphosphatealdolase | fbpA_F1^b^  fbpA_R1^b^ | GCTGCTCCRCTTGGYWTGAT  CCRCCAGARAAAAYYACTATTC | 900 | 429 | Baldo et al. 2006 [41] |
| WD_1063 | *wsp* | Outer surface protein | wsp_F1  wsp_R1 | GTCCAATARSTGATGARGAAAC  CYGCACCAAYAGYRCTRTAAA | 714 | 513 | Baldo et al. 2006 [41] |
| WD_1063 | *wsp* | Outer surface protein | 81F^b^  691R^b^ | TGGTCCAATAAGTGATGAAGAAAC  AAAAATTAAACGCTACTCCA | 714 | 513 | Braig et al. 1998 [57] |
| WD_Wp16SA | *16S rRNA* | 16S ribosomal RNA | wspecF^b^  wspecR^b^ | YATACCTATTCGAAGGGATAG  AGCTTCGAGTGAAACCAATTC | 1,447 | 438 | Werren& Windsor 2000 [2] |
|  | *12S rRNA* | mitochondrial 12S ribosomal RNA | 12SCFR  12SCRR | GAGAGTGACGGGCGATATGT  AAACCAGGATTAGATACCCTATTAT |  | 380 | Hanner & Fugate  1997 [55] |

^a^ With respect to the *w*Mel genome.

^b^ Used for the amplification of both cytoplasmic and chromosomal Wolbachia gene fragments.
