# Supplementary figures and images for: Detection and characterization of Wolbachia infections in laboratory and natural populations of different species of tsetse flies (genus Glossina)
Source: BMC Microbiol. 2012 Jan 18;12(Suppl 1):S3. doi: 10.1186/1471-2180-12-S1-S3 (PMC3287514; doi:10.1186/1471-2180-12-S1-S3)

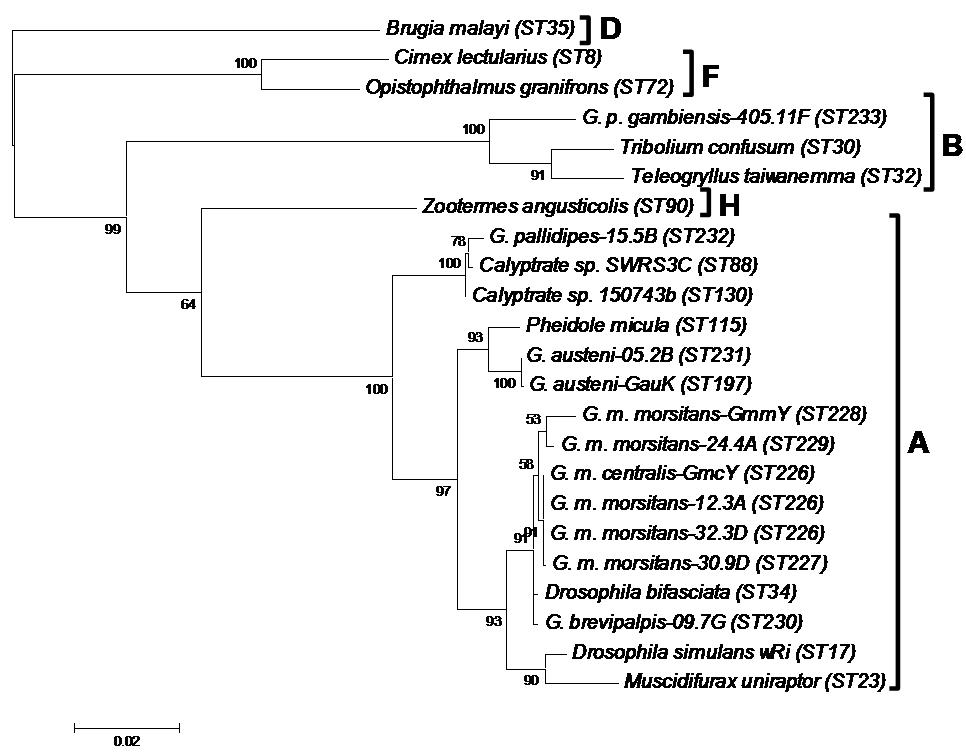

Supplement: Additional file 2 — Supplementary Figure 1: Maximum likelihood inference phylogeny based on the concatenated MLST data, 2,079 bp. (Please note that tree has been rooted to the supergroup D sequences). [file 1471-2180-12-S1-S3-S2.jpg]

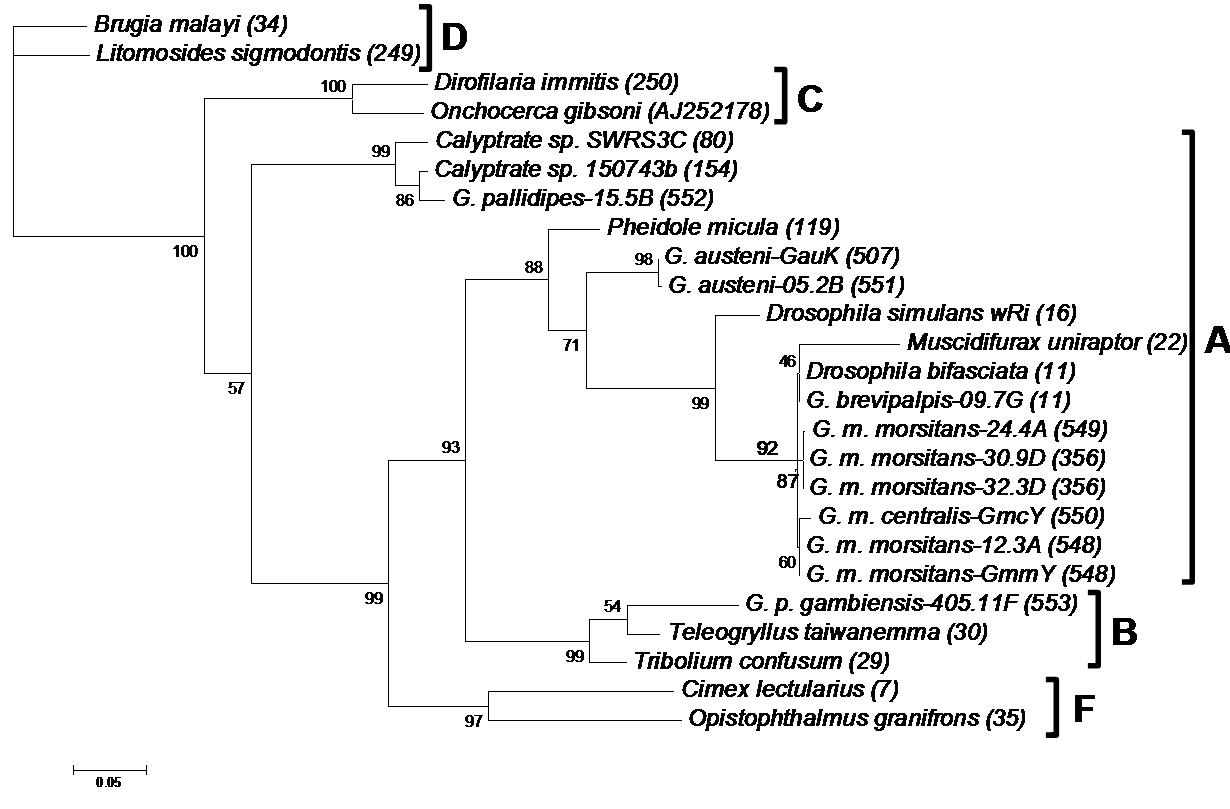

Supplement: Additional file 3 — Supplementary Figure 2: Maximum likelihood inference phylogeny based on the on the wsp sequence. (Please note that tree has been rooted to the supergroup D sequences). [file 1471-2180-12-S1-S3-S3.jpg]
